# Brown Adipose Tissue (BAT) Causing Unusual Cervical and Scapular Uptake of ^18^F-FDG in a Young Patient with Hodgkin’s Lymphoma

**DOI:** 10.5334/jbr-btr.830

**Published:** 2015-09-15

**Authors:** B. Coulier, L. Montfort, F. Richelle, Ch. Brichant

**Affiliations:** 1 Department of Diagnostic Radiology, Clinique St-Luc, Bouge (Namur), Belgium; 2 Department of Internal Medicine, Clinique St-Luc, Bouge (Namur), Belgium; 3 Department of Nuclear Medicine, Clinique St-Luc, Bouge (Namur), Belgium; 4 Department of Diagnostic Radiology and Nuclear Medicine, Clinique St-Luc, Bouge (Namur), Belgium

A 23-year-old Tunisian medical student was referred to our department of nuclear medicine for ^18^F-FDG PET/CT post treatment evaluation of a grade 3 mediastinal Hodgkin’s lymphoma. PET/CT was unavailable in Tunisia and the patient came especially to Belgium where he had family. He had been treated with eleven courses of chemotherapy comprising cyclophosphamide, adriamycin, etoposide and corticosteroids.

PET/CT demonstrated a residual mass in the superior and anterior mediastinum – the thymic loge – but there was no residual metabolic activity suggesting a complete remission of the disease (white arrow on Fig. B). However, intense FDG uptake was demonstrated symmetrically in the scapulars and cervical areas of the patient (black arrows on Fig. A). These areas exclusively showed fat attenuation on the corresponding CT images (Fig. B) and were diagnosed as typical of Brown Adipose Tissue or BAT-related FDG uptake. The patient was young, had a very low body mass index and had undoubtedly been submitted to a colder environment in Belgium, all circumstances which are known as determinants circumstances of the prevalence, mass, and activity of ^18^F-FDG-detected BAT.

**Figures A–B:**
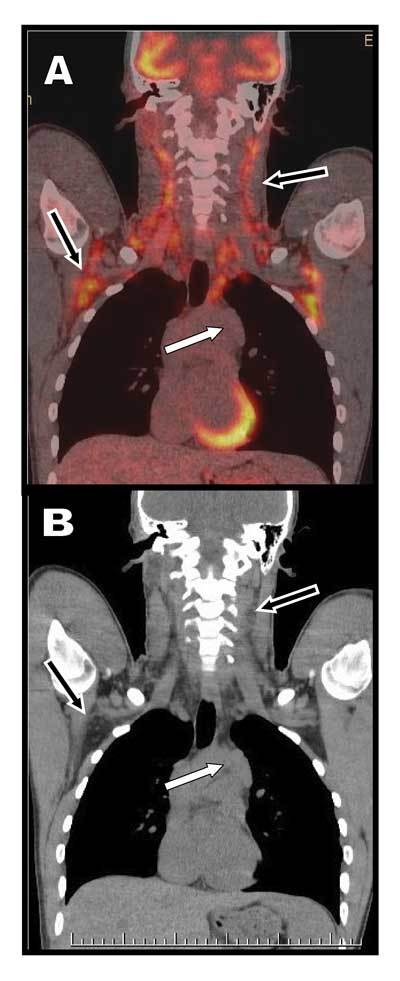


## Comment

Positron Emission Tomography /Computed Tomography (PET/CT) is an efficient imaging modality for the staging and restaging of patients with Hodgkin’s and non Hodgkin’s lymphoma (NHL). The sensitivity of PET is 83% for pathologic peripheral lymph nodes, 91% for thoracic lymph nodes and 75% for abdominal and pelvic lymph nodes.

Nevertheless non-neoplastic physiologic or pathologic processes can also be associated with increased PET/CT fluodesoxyglucose (FDG) uptake. Among these, Brown Adipose Tissue (BAT) detection rate is known being high with a cold outdoor temperature (winter time), a younger age (BAT decreasing with age and especially abruptly after age 40 years), female sex and a low body mass index.

BAT is a energy-expanding tissue that generates heat in response to exposure to cold (nonshivering thermogenesis) and that particularly in women but also in response to ingestion of food (diet-induced thermogenesis). BAT has a rich vascular supply, is rich in sympathetic nerves and may have a high metabolic activity. Thus under sympathetic stimulation BAT may have an intense FDG uptake. Spontaneous physiological FDG positive BAT is variable ranging from 1,72% to 6,85% in studies involving large cohorts of cancerous patients who undergo PET/CT. BAT decreases when obesity increases but the true relation between BAT and obesity is not clearly confirmed. Nevertheless there is a great excitement about the potential for harnessing BAT to treat obesity or diabetes mellitus.

The most common sites of BAT are the supraclavicular areas, the ventral neck area but also the paravertebral, axillary, mediastinal and sometimes upper abdominal areas. A symmetrical distribution is the rule and facilitates the diagnosis.

A typical finding of BAT-related FDG uptake is symmetrical FDG uptake in the supraclavicular, mid-axillary, paraspinal and posterior mediastinal regions. These areas show fat attenuation (–50 to –150 Hounsfield units) on the corresponding CT images. Precise PET/CT image fusion, careful analysis of CT images and knowledge of human BAT distribution help to avoid misdiagnosis of brown fat as deposits for pathology. Sedatives which have been found as a helpful tool to reduce unspecific FDG muscle uptake are ineffective to reduce atypical uptake of brown fat.

## Competing Interests

The authors declare that they have no competing interests.
